# Lumbar spine DXA *T*-score and QCT BMD cutpoint values for defining osteofrailia among older men: a framework for further refinement

**DOI:** 10.1007/s00256-026-05133-2

**Published:** 2026-01-23

**Authors:** Yì Xiáng J. Wáng, Timothy C. Y. Kwok, Maria Pilar Aparisi Gómez, Ben-Heng Xiao, Jason C. S. Leung, Fernando Ruiz Santiago, Wing P. Chan, Daniele Diacinti, Ali Guermazi, Alberto Bazzocchi

**Affiliations:** 1 Department of Imaging and Interventional Radiology, Faculty of Medicine, The Chinese University of Hong Kong, Shatin, Hong Kong, SAR China; 2 Jockey Club Centre for Osteoporosis Care and Control, Faculty of Medicine, The Chinese University of Hong Kong, Shatin, Hong Kong, SAR China; 3 Department of Medicine and Therapeutics, Faculty of Medicine, The Chinese University of Hong Kong, Shatin, New Territories, Hong Kong, SAR China; 4 Department of Radiology, Auckland City Hospital, Auckland District Health Board, Auckland, New Zealand; 5 Department of Anatomy and Medical Imaging, Faculty of Medical and Health Sciences, The University of Auckland, Auckland, New Zealand; 6 Department of Radiology, IMSKE, Valencia, Spain; 7 Department of Radiology and Physical Medicine, Faculty of Medicine, University of Granada, Granada, Spain; 8 Musculoskeletal Radiology Unit, Hospital Universitario Virgen de Las Nieves, Granada, Spain; 9 Department of Radiology, Wan Fang Hospital, Taipei Medical University, Taipei, Taiwan; 10 Department of Radiology, School of Medicine, College of Medicine, Taipei Medical University, Taipei, Taiwan; 11 Department of Radiological Sciences, Oncology and Pathology, Sapienza University of Rome, Rome, Italy; 12 Department of Radiology, Boston University School of Medicine, Boston, MA USA; 13 Diagnostic and Interventional Radiology, IRCCS Istituto Ortopedico Rizzoli, Bologna, Italy

**Keywords:** Osteoporosis, Bone mineral density (BMD), *T*-score, Males, Quantitative computed tomography

## Abstract

Older men suffer from hip FFx (fragility fracture) at femoral neck *T*-score approximately 0.6 higher than older women, thus we proposed a new category of low BMD status, osteofrailia, for older Caucasian men with femoral neck *T*-score ≤ −2.0 (*T*-score ≤ −2.1 for older East Asian men) who have an increased risk of hip FFx. Around the age of 78 years, mean LS (lumbar spine) QCT BMD is around 68 mg/mL and 100 mg/mL for East Asian men and Caucasian men, respectively. For East Asian men, LS QCT BMD <68 mg/mL offers a sensitivity of 77% for detecting vertebral FFx cases, which is consistent with LS QCT BMD <80 mg/mL and < 50 mg/mL offering a vertebral FFx detection sensitivity of around 77% for Caucasian women and East Asian women, respectively. For Chinese men, *T*-score ≤ −2.5 predicts hip FFx risk better than other *T*-score values, and LS DXA *T*-score − 2.5 corresponds to QCT BMD 68 mg/mL. Hip FFx occur at approximately 0.5 LS *T*-score higher in Caucasian men than in Caucasian women. Among older Caucasian populations, for the separation of patients with FFx and without FFx, QCT BMD <100 mg/mL in older men is approximately comparable to <80 mg/mL in older women. For FFx risk prediction, we propose osteofrailia threshold LS DXA *T*-score to be ≤ − 2.5 and ≤ −2.0, and QCT BMD to be <68 mg/mL and < 100 mg/mL, respectively, for East Asian men and Caucasian men. The relationship between LS QCT BMD and hip FFx risk should be better investigated in the future.

Operational ranges of Dual-Energy X-ray Absorptiometry (DXA) *T*-score were initially proposed for epidemiological classification purposes. When the femoral neck (FN) BMD is measured in adult Caucasian women, a cutpoint value of 2.5 standard deviation (SD) below the young adult mean results in a prevalence of osteoporosis for those aged ≥50 years of about 16.2%, the same as the lifetime risk of hip Fragility fracture (FFx) for Caucasian women. Densitometric osteoporosis prevalence among a specific population should be in proportion to its relative osteoporotic Fx risk, with Caucasian female data as reference [1]. Both Caucasian and East Asian men suffer from hip FFx at a FN *T*-score approximately 0.6 higher than women, thus we proposed a new category of low BMD status, osteofrailia, as a FN *T*-score ≤ −2.0 for older Caucasian men likely to suffer from hip FFx (FN *T*-score ≤ −2.1 for older East Asian men) [2, 3]. It is noted that osteofrailia defined by FN *T*-score is associated with these three features [2]: 1) prevalence of osteofrailia (inclusive of osteoporosis) in men is similar to prevalence of osteoporosis in women; 2) hip FFx risk among osteofrailiac men is approximately half of that of osteoporotic women; 3) mean hip FFx FN *T*-score is approximately 0.5 lower than the FN osteofrailia cutpoint value, thus, FN osteofrailia cutpoint value is suitable for hip FFx risk screening in older men. That osteofrailia classification is tied to the FFx prevalence of the population is relevant so that, an osteofrailia diagnosis among different populations can have the same meaning thus allowing international comparison. Literature analysis shows that, by applying the appropriate hip DXA osteoporosis threshold for women and osteofrailia threshold for men, approximately 70% of incident hip FFx can be predicted for both older women and men [3]. However, compared with that of women, men’s hip FFx is still less predictable with studies reporting larger SDs for men’s mean hip FFx FN *T*-score [2].

While we established the FN *T*-score cutpoint value for defining osteofrailia among older men with strong evidence [2], the lumbar spine (LS) *T*-score for defining osteofrailia among older men has not been studied. When a DXA scan is conducted, the FN *T*-score is more relevant than LS *T*-score, given FN *T*-score predicts hip FFx better than LS T-score, and discordance between LS *T*-score and FN *T*-score is common [4]. Recently, with the increased availability of CT scan and quantitative CT (QCT) based densitometry, spine QCT BMD, rather than FN BMD, is often ‘opportunistically’ reported for older patients when the primary scan target is the lung or abdominal organs [5, 6]. QCT for BMD measurement can be performed on any CT scanner with the use of a calibration phantom and dedicated analysis software. Compared with DXA, QCT is less susceptible to pitfalls caused by degenerative changes, as it is a non-projectional technique, able to specifically measure bone density of the vertebral body, excluding osteophytes and facet joint degeneration as well as soft tissue calcifications. QCT is able to separately measure the density of trabecular bone, more sensitive to detect small changes in the context of certain diseases and therapies [7]. Disadvantages of QCT include higher effective radiation dose and patient exposure and less data on fracture risk prediction compared to DXA. Based on a limited number of studies on female patients which mainly correlated spine trabecular QCT BMD and spine fractural deformity on radiographs, the cutpoint value for classifying osteoporosis was recommended as <80 mg/mL QCT BMD [8]. Despite the primary studies were conducted mainly on Caucasian women, this cutpoint value was applied to men as well [9]. To adjust for the lower FFx prevalence and the lower normative BMD values among East Asians, we recommended that for East Asians, LS trabecular QCT BMD osteoporosis cutpoint value was adjusted to <50 mg/mL [10–12].

Although the initial definition of densitometric osteoporosis has an epidemiological classification purpose, opportunistic reporting of QCT BMD is aimed towards FFx risk prediction. While the LS trabecular QCT BMD cutpoint values for defining osteoporosis among women (i.e., <80 for Caucasians and < 50 mg/mL for East Asians) are likely suitable for FFx risk screening purposes [8, 10, 11], the LS QCT BMD cutpoint value for defining osteoporosis among Chinese men (i.e., also <50 mg/mL) was defined as an epidemiology concept [12], and not suitable for the purpose of FFx risk screening. The LS QCT BMD cutpoint value for FFx risk screening among Caucasian men has also not been determined. In this article, based on currently available evidence, we propose LS *T*-score and QCT cutpoint values for defining osteofrailia among older Chinese and Caucasian men that can serve the purpose of screening FFx risk. It should be noted the values and numbers described in this article are only estimations, we believe with more work and more data we can further refine the recommendations in the future. An optimal osteoporosis BMD screen threshold for women and osteofrailia BMD screen threshold for men will be particularly useful for ‘case-finding’ of FFx high risk subjects at an ‘earlier’ age.

## An introduction to the FN osteofrailia *T*-score criterion

**Fig. 1:**
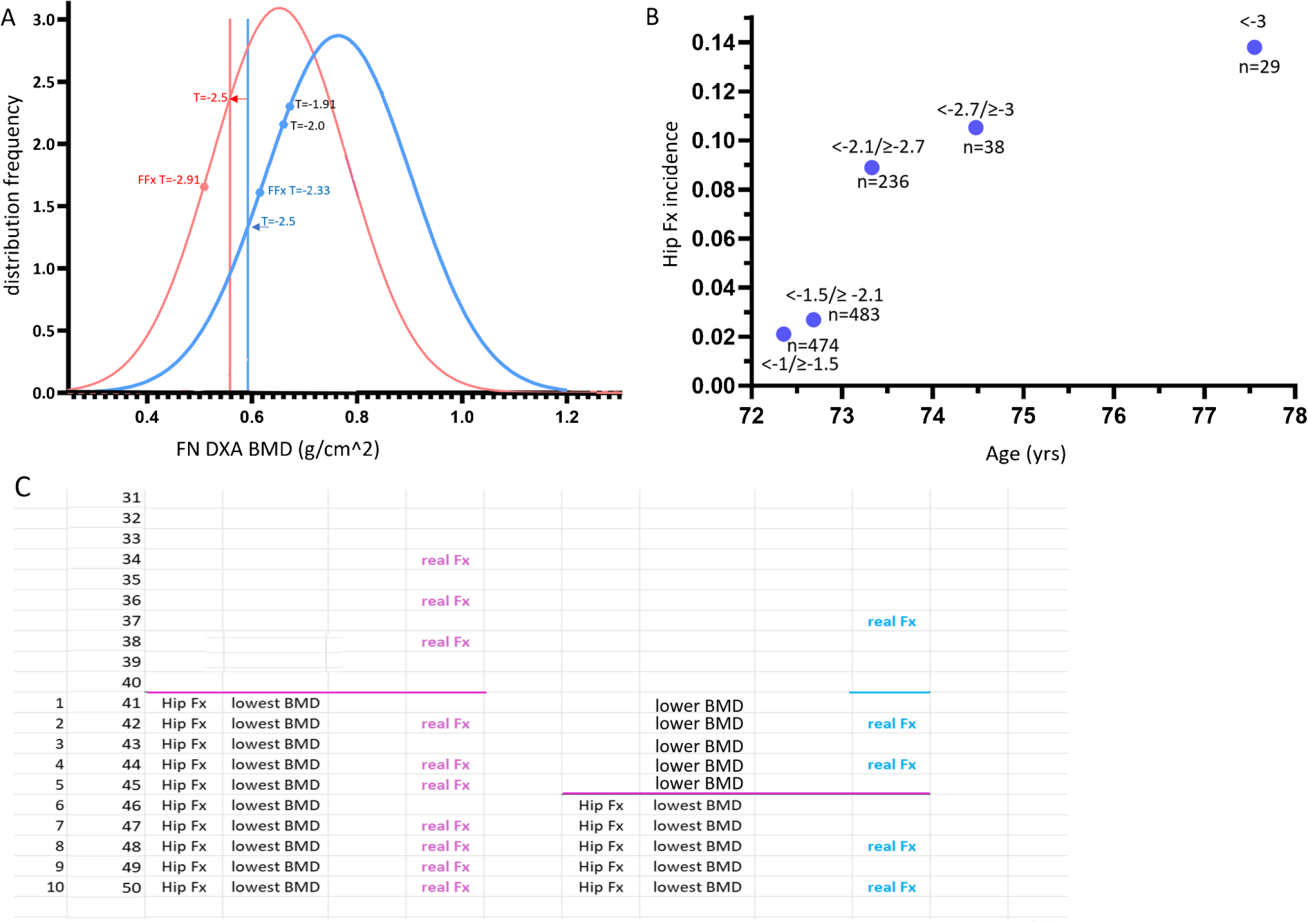
**A**: The femoral neck (FN) BMD distribution of US Caucasians and thresholds to define osteoporosis. The BMD distribution data of older population (≥50 years) are from Looker et al. [13]. Red arrow: the *T*-score to define osteoporosis in women. Blue arrow: the *T*-score to define osteoporosis in men. *T*-score = −2.91 and *T*-score = − 2.33 are the approximately estimated mean hip fracture *T*-scores for women and men respectively [2]. **B**: Hip FFx incidence  (Y-axis, %) among older Chinese men associated with various groupings of baseline FN *T-*score (blue dots). Data are from MrOS Hong Kong study follow-up results for males (*n* = 1260 cases with FN *T*-score < −1). X-axis is the mean age for each FN *T*-score grouping. In an observational manner, male participants were followed up for a total of 9.9 ± 2.8 years, and 63 hip FFx (mean fracture age: 82.5 ± 5.7 years) were recorded (dropouts have not been adjusted in the graph). The data show that when baseline FN *T*-score was higher than −2.1, then hip FFx incidence was ‘*low*’ during the follow-up period. After the baseline FN *T*-score was lower than −2.1, hip FFx incidence increased substantially. After the FN *T*-score was lower than −2.7, hip FFx incidence increased further but only relatively slightly [3]. **C**: An illustration of osteofrailia concept. Assuming the hip FFx prevalence among a female population is 20%, then in 50 women as a presentative sample, 10 cases would suffer from hip Fx. Among them, the 10 cases with the lowest hip BMD are classified as being osteoporotic; and among the 10 cases, 7 would develop hip FFx. If we use the osteoporosis threshold, then 70% of them could be predicted, while another 30% could not be predicted. Men’s hip FFx prevalence is approximately half of that of the women, thus in 50 men as a presentative sample, 5 cases would suffer from hip FFx. Among these 50 men, the 5 cases with the lowest hip BMD are classified as being osteoporotic. However, 3 of the hip FFx cases have a BMD higher than the osteoporosis threshold (pink line). When osteofrailia threshold (blue line) is applied, then 10 cases with low hip BMD are included (5 cases with lower BMD and 5 cases with lowest BMD). Only 1 of the hip FFx cases has a BMD higher than the osteofrailia threshold. Osteoporotic women and osteofrailiac men both have a prevalence of 20%

The clinical significance of osteoporosis lies in the occurrence of FFx. The most relevant FFx site is the hip, and hip FFx typically requires hospitalization thus data on incidence are more reliable. The rational for the osteofrailia threshold is explained in Fig. 1. Figure 1A illustrates the DXA BMD distribution of USA Caucasians data based on the report by Looker et al. [13]. Caucasian older men suffer from hip FFx at 0.6 FN *T-*score higher than women (which parallels East Asian data). In Fig. 1A, while the mean female hip FFx FN *T*-score of −2.91 lies substantially below the female osteoporosis *T*-score threshold, which is reasonable from a FFx risk screening point of view, the mean male hip FFx FN *T*-score of −2.33 lies above the male osteoporosis *T*-score threshold, suggesting using the FN *T*-score ≤ −2.5 is not practical for FFx risk screening amongst men. The FN *T*-score of approximately ≤ − 2.0 (i.e., the osteofrailia threshold) is a more suitable cutpoint value to identify high risk of FFx in older Caucasian men [2, 3]. Accordingly, we recommended using an osteofrailia FN *T*-score of ≤ − 2.1 to detect high risk of FFx in older East Asian men [2, 3].

For older Chinese men, the osteofrailia FN *T*-score cutpoint (i.e., −2.1 rather than −2.5 or − 2.7) appears to be a natural threshold for a higher hip FFx risk (Fig. 1B) [3]. Data in Fig. 1B are follow-up results from the MrOS Hong Kong study (*n* = 2000 at baseline). Male participants were followed up for a total of 9.9 ± 2.8 years, and 63 hip FFx were recorded (mean fracture age: 82.5 ± 5.7 years). When the baseline FN *T*-score used is higher than −2.1, hip FFx incidence is ‘*low*’ during the follow-up period. If the baseline FN T-score used is lower than −2.1, hip FFx incidence increases substantially. If we use a FN *T*-score ≤ −2.7 in male study subjects, then only a small portion of hip FFx cases are predicted during the 9.9-year follow-up (15.9%, 10/63). If we use a FN *T*-score ≤ −2.1, which is equivalent to the Caucasian male *T*-score of −2.0, then 46.0% (29/63) of hip FFx cases can be predicted in male study subjects. For female participants, if we use a *T*-score ≤ −2.7 (equivalent to the Caucasian female *T*-score of −2.5) to determine study subjects, then 44.9% (31/69) of hip FFx cases could be predicted during the 8.8 years’ follow-up [2].

Evidence suggests that, for both East Asians and Caucasians, the prevalence of hip FFx in older women is approximately twice than that of older men [14–17]. Thus, the prevalence of osteoporosis in older men would be approximately half of that in older women. A FN *T*-score cutpoint of ≤−2.5 in older women defines 23.4% of the US older Caucasian female population [2, 13]. If we use a FN *T*-score ≤ −2.0 to define osteofrailia, then osteofrailia prevalence in older US Caucasian men would be 22.7% [2]. In the MrOS and MsOS Hong Kong studies, a FN *T*-score of ≤ − 2.7 defines 16.9% of the Chinese women being osteoporotic (mean age: 72.5 yrs), and a FN *T*-score of ≤ − 2.1 defines 15.5% of the Chinese men being osteofrailiac (mean age: 72.3 years) [2].

## If a LS *T*-score ≤ −2.5 criterion is defined as osteofrailia, then LS osteofrailia prevalence among Chinese men is similar to the LS osteoporosis prevalence among Chinese women

Osteoporosis prevalence in Chinese women is approximately half of that of Caucasian women [18, 19]. The prevalence of vertebral FFx among East Asian women is not more than half of that in Caucasian women [20, 21]. When a LS *T*-score of ≤ − 3.7 is used to define osteoporosis, osteoporosis prevalence for Hong Kong Chinese women is around 8% [18]. From the epidemiological point of view, LS DXA *T*-score criterion for defining osteoporosis among older Chinese men has been proposed to be <−3.2 [18, 19, 22]. Since the FN osteoporosis *T*-score cutpoint is 0.6 lower than for osteofrailia FN *T*-score cutpoint (i.e., −2.7 vs −2.1), we can tentatively suggest osteofrailia LS *T*-score cutpoint to be ≤ − 2.5 [3]. When LS osteofrailia *T*-score criterion is ≤ − 2.5 among older Chinese men, the LS osteofrailia prevalence is also around 8% (8.27% for Hong Kong data and 8.32% for China mainland data [18, 23]), which is similar to the LS densitometric osteoporosis prevalence for Chinese women (Table 2 in [18]). Therefore, from an epidemiological point of view, the osteofrailia criterion of LS *T*-score ≤ −2.5 is appropriate for Chinese men.

## For Chinese men, a LS *T*-score of ≤ − 2.5 is a better risk predictor for hip FFx than other LS *T*-score values

In the MrOS Hong Kong study, the study subjects (*n* = 2000 at baseline) had a baseline mean age of 72.3 years (range, 65–92 years), and they were followed up for 9.9 ± 2.8 years and 63 hip FFx were recorded. Positive predictive value was the percentage hip FFx cases out of the total tested positive cases at baseline by a metric. Detection sensitivity was the percentage tested positive cases at baseline by a metric out of the total hip FFx cases. The LS *T-*scores of ≤ − 2.5 and ≤ −2.2 offered a positive predictive value of 8.85%, 6.86%, and detection sensitivity of 27.0%, 30.2% respectively [3]. LS *T-*score of ≤ − 2.5 offers a detection sensitivity comparable to *T-*scores of ≤ − 2.2, while with a better positive predictive value.

On a five-year follow-up, a total of 23 hip FFx were observed. The LS *T*-scores of ≤ − 2.7, ≤ − 2.5, and ≤ −2.3 offered a positive predictive value of 5.303%, 5.0%, and 4.310%, and a detection sensitivity of 30.4%, 39.1%, and 43.5% respectively [24]. LS *T*-score of ≤ − 2.5 offers a positive predictive value close to LS *T*-score of ≤ − 2.7 and a detection sensitivity close to LS *T*-score of ≤ − 2.3, representing a better compromise. Thus, the LS *T*-score of ≤ − 2.5 is the favored cutpoint value for hip FFx prediction among Chinese men, over the cutpoint value of ≤ − 2.7, ≤ − 2.3, or ≤ −2.2 [24, 25].

## If the criteria of DXA LS *T*-score ≤ −2.5 (osteofrailia) and BMD <68 mg/mL on LS QCT are used, at age 78, half of Chinese men are osteofrailiac

The osteofrailia LS *T*-score to be ≤ − 2.5 for Chinese men corresponds to a LS DXA BMD ≤0.715 g/cm^2^ in a Hologic densitometer [18]. Correlation analyses show LS Hologic densitometer DXA BMD 0.715 g/cm^2^ is equivalent to LS QCT BMD of 68 mg/mL [24].

**Fig. 2:**
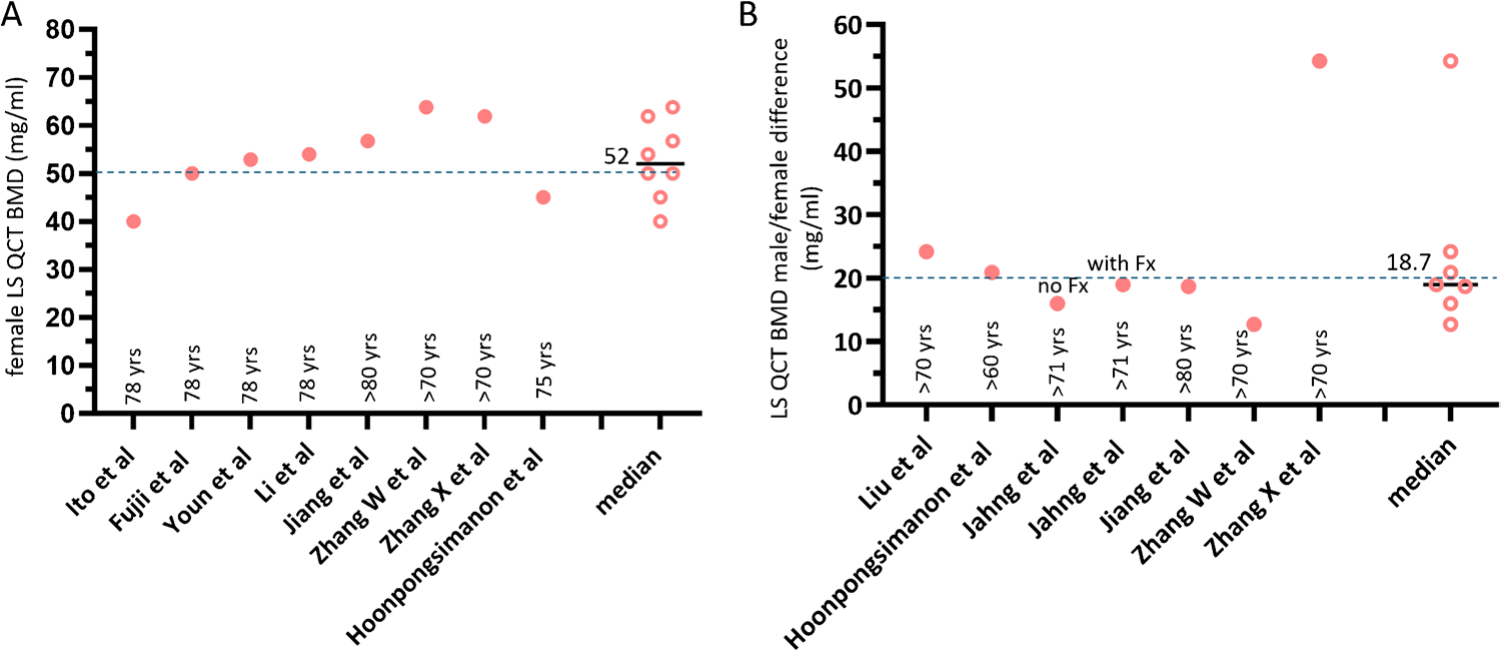
Women’s LS QCT BMD at the age of around 78 years (**A**, dotted blue line: value of 50), and difference of such a BMD between men and women (**B**, dotted blue line: value of 20). Data are from East Asia and Thailand. It is noted that, at the age of around 78 years, mean LS QCT BMD is around 50 mg/mL for women, and men’s BMD is approximately 18.7 mg/mL higher. Data are from: Ito et al. Calcif Tissue Int 1997;61:123–8; Fujii et al. Bone Miner 1989;6:87–94. Youn et al. J Korean Soc Spine Surg 2006;13:255–61; Li et al. Chin J Osteopros. 2019;25:1257–1272; Jiang C, et al. J Med Imaging 2016; 26: 1278–1283 (in Chinese); Zhang W, et al. Endocrine 2014;47:862–8. Zhang X et al. Arch Osteoporos. 2019;14:31; Hoonpongsimanon et al. J Med Assoc Thai 2005;88:1666–73; Liu et al., Front Endocrinol (Lausanne) 2022;13:1013597; Jahng et al. J Korean Orthop Assoc. 1990; 25:262–269 (including data with and without radiographic vertebral Fx). The data of Jiang et al., Zhang W et al., and Zhang X et al. are measured results, others are fitted results. Median: median of the listed values

It has been consistently shown that, at the age 78 years old, the mean LS QCT BMD for Caucasian women is around 80 mg/mL [10, 11]. Thus, considering LS QCT BMD is <80 mg/ml as the threshold for osteoporosis, at 78 years old, half of Caucasian women are densitometrically osteoporotic. It has been consistently shown that, at the age 78 years old, the mean LS QCT BMD for East Asian women is around 50 mg/mL (Fig. 2A). At this age, half of the Chinese women are also densitometrically osteoporotic.

Figure 2B shows, at the age of around 78 years, Chinese men have a LS QCT BMD that is 18.7 mg/mL higher than women. Thus, at 78 years, if the mean LS QCT BMD cutpoint value is <68 mg/mL (approximately = 50 + 18.7 mg/mL), half of the Chinese men are osteofrailiac. This is consistent with the concept that the prevalence of osteofrailia among older men and prevalence of osteoporosis among older women are similar.

## East Asian male patients suffer from vertebral FFx at a LS QCT BMD approximately 18.5 mg/mL higher than female patients

**Fig. 3:**
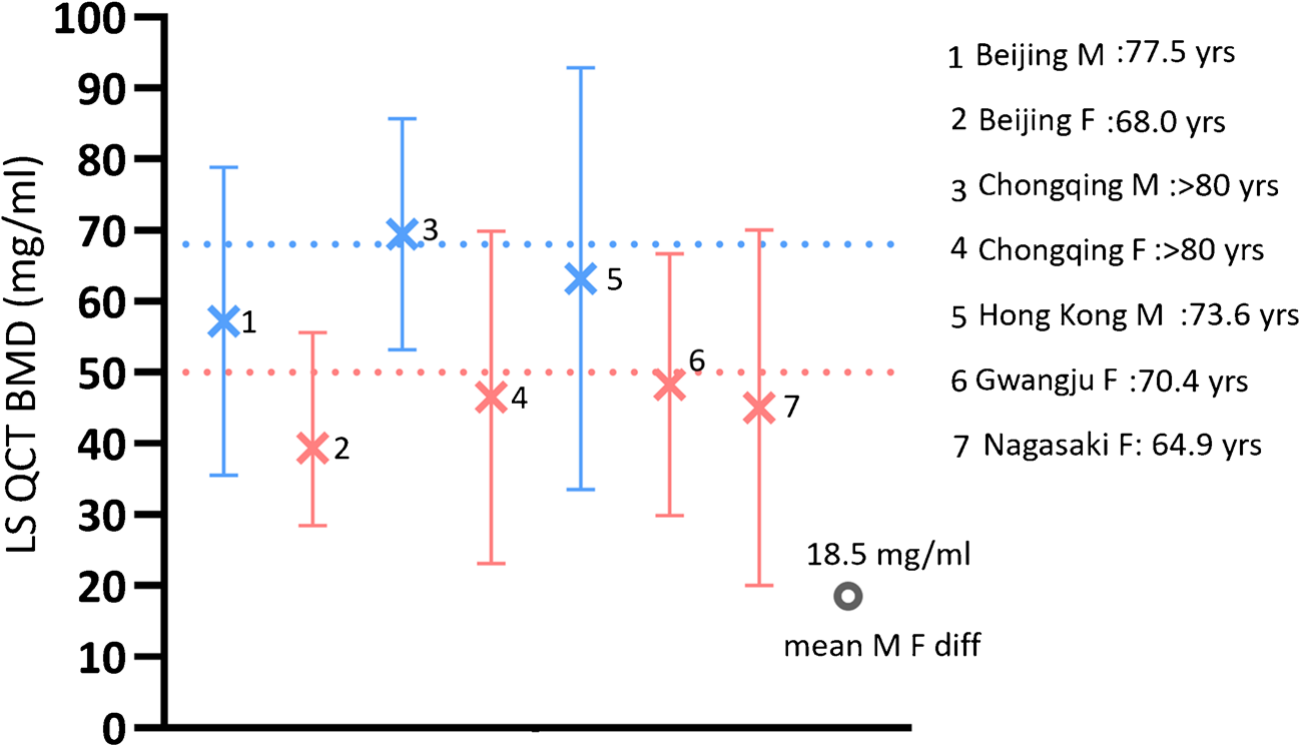
Among older East Asians, male patients suffer from vertebral FFx a LS QCT BMD approximately 18 mg/ml higher than female patients. Dote blue line: value of 68 mg/ml, dote red line: value of 50 mg/ml. Data source, 1 and 2: clinical low energy trauma Fx (Beijing, data from: Mao et al. J Orthop Translat. 2018;16:33–39); 3 and 4: Low energy trauma caused fresh Fx < 1 week [Chongqing, data from Jiang et al. J Med Imaging (In Chinese) 2016; 26: 1278–1283]; 5: Hong Kong males’ data from MrOS study, radiographic Fx is defined as OLVFss ≤ − 2.5 [24]. 6. radiographic Fx [Gwangju, data from: Youn et al. J Korean Soc Spine Surg 2006;13:255–61]; 7. radiographic Fx (Nagasaki, data from: Ito et al. Calcif Tissue Int 1997;61:123–8)

It has been noted that male patients suffer from FFx at a higher BMD than female patients. This is valid for the spine and for the hip [2, 25]. Fig. 3 shows that East Asian male patients suffer from vertebral FFx at a LS QCT BMD approximately 18.5 mg/mL higher than female patients.

**Fig. 4:**
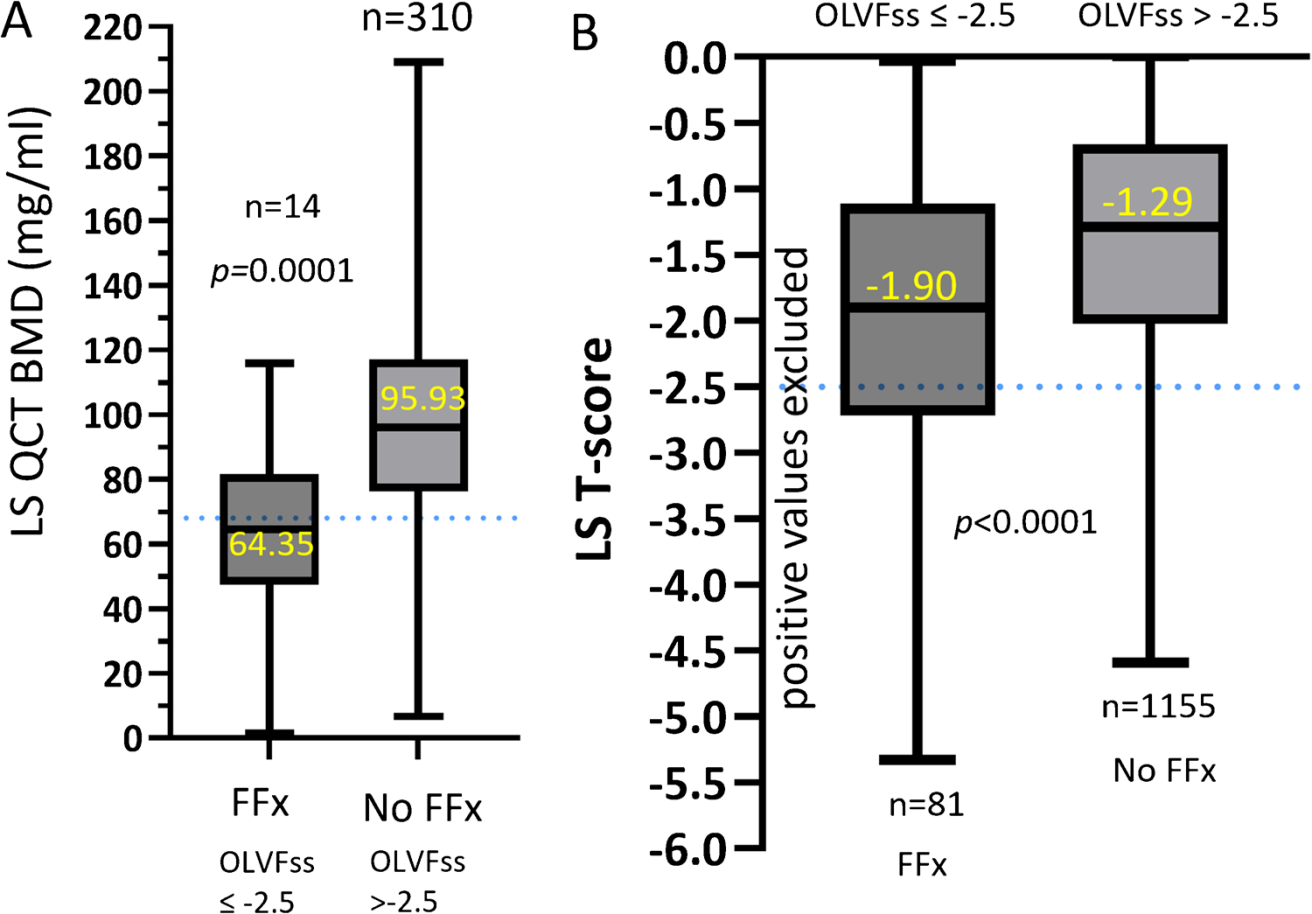
Relationship between OLVFss and LS BMD. QCT BMD identifies subjects with vertebral FFx better than DXA *T*-score. **A** shows subjects with vertebral FFx defined as OLVFss ≤ − 2.5 have a median QCT BMD lower than 68 mg/ml (threshold denoted by the blue dotted line), data from MrOS Hong Kong study year-2 follow-up. **B**: MrOS Hong Kong study baseline data with positive LS DXA *T*-sore subjects excluded. Subjects with vertebral FFx and without vertebral FFx had LS *T*-score median value of −1.90 and − 1.29 respectively, these values are higher than *T-*score of −2.5 (blue dotted line). Data are shown with box and whiskers plot

An osteoporotic-like vertebral fractural deformity (OLVF) sum score (OLVFss) has been proposed as a metric to assess vertebral bone strength [24, 26–28]. In brief, on spine imaging, vertebrae L3 to L5 are evaluated with an extended version of a semi-quantitative scheme for vertebral fractural deformity [26]. For each vertebra in a subject, a score of 0, −0.5, −1, −1.5, −2, −2.5, and − 3 is assigned for no-OLVF or OLVF of <20%, ≥20 ~ 25%, ≥25% ~ 33%, ≥33% ~ 40%, ≥40% ~ 66%, and ≥ 66% vertebral height loss, respectively. The OLVFss is calculated by adding up the scores of vertebrae T3 to L5, while two adjacent minimal OLVF are scored as −0.5. We showed that OLVFss ≤ − 2.5 suggest vertebral FFx for Chinese men [24, 25, 28]. Our data showed that LS QCT BMD of 68 mg/mL offers a sensitivity for radiographic vertebral FFx of 77% (AUROC of 78.9%) for the discrimination of those with OLVFss ≤ − 2.5 and those with OLVFss > − 2.5 (Fig. 4A) [24]. This is consistent with LS QCT BMD <80 mg/mL offering a vertebral FFx detection sensitivity of around 77% for Caucasian women [8], and BMD <50 mg/mL offering a vertebral FFx detection sensitive of around 77% for Chinese women [10, 11, 29].

However, Fig. 4B shows that the majority of Chinese men with OLVFss ≤ − 2.5 (i.e., osteofrailiac range) have LS DXA > −2.5 (i.e., not osteofrailiac range). This is in agreement with the report that LS *T*-score is often artificially inflated due to spine degenerative changes and thus insensitive to diagnose low BMD status in the LS, and QCT BMD is more sensitive in predicting vertebral FFx risk [30–32]. The equivalent OLVFss cutpoint for osteoporosis in Chinese women is ≤ − 1.5, and OLVFss cutpoint for osteofrailia in Chinese men is ≤ − 2.5 [24, 28]. OLVFss in men is also less sensitive for hip Fx prediction than OLVFss in women [3]. In the MsOS and MrOS Hong Kong studies, female participants were followed up for 8.8 ± 1.5 years, and 69 hip FFx were recorded; male participants were followed up for 9.9 ± 2.8 years, and 63 hip FFx were recorded, respectively. For female participants, osteoporosis defined by OLVFss had a prediction sensitivity of 43.5% (30/69), being very similar to the prediction sensitivity of FN BMD defined osteoporosis of 47.8% (33/69). On the other hand, OLVFss in men with cutpoint value of ≤ − 2.5 only had prediction sensitivity of 27.0% (27/63), being inferior to that of FN BMD defined osteofrailia 46.0% (29/63) [3].

As a summary of what has been discussed in sections 2, 3, 4, and 5, we can confidently recommend that, for Chinese men, LS osteofrailia *T*-score is ≤ − 2.5, and osteofrailia LS QCT BMD is <68 mg/mL (Table 1).

**Table 1:** Observations and results for supporting osteofrailia criterion in Chinese men

Osteofrailia criterion	Supporting observations and results	Evidence strength
LS DAX *T*-score cutpoints value ≤ − 2.5	LS *T*-score of ≤ − 3.7 is used to define osteoporosis, osteoporosis prevalence for Chinese women is around 8%; when LS *T*-score criterion is ≤ − 2.5, osteofrailia prevalence for Chinese men is also around 8%.	strong
	LS *T*-score of ≤ − 2.5 is the favored cutpoint value for hip FFx prediction, over the cutpoint values of ≤ − 2.7, ≤ − 2.3, or ≤ −2.2.	modestly strong
	East Asian men's hip FFx occur at 0.5 LS *T*-score higher than East Asian women	weak
LS QCT BMD cutpoints value <68 mg/ml	Around 78 years old, the mean LS QCT BMD is 50 mg/ml for East Asian women, half of the women are densitometrically osteoporotic; at this age, if the cutpoint LS QCT BMD is <68 mg/ml, half of the Chinese men are osteofrailiac.	modestly strong
	If LS osteofrailia *T*-score is ≤ − 2.5 for Chinese men, then Hologic densitometer BMD is ≤0.715 g/cm^2^ for Chinese men, and DXA BMD of 0.715 g/cm^2^ correlates to QCT BMD of 68 mg/ml.	modestly strong
	Male patients suffer from vertebral FFx at a LS QCT BMD approximately 18.5 mg/ml higher than female patients.	modestly strong
	LS QCT BMD <68 mg/ml offers a vertebral FFx detection sensitivity of 77%. This is consistent with BMD <80 mg/ml offering a vertebral FFx detection sensitivity of around 77% for Caucasian women, and BMD <50 mg/ml offering a vertebral FFx detection sensitivity of around 77% for Chinese women.	modestly strong

## Hip FFx occur at approximately 0.5 higher LS *T*-score in Caucasian men than in Caucasian women

**Fig. 5:**
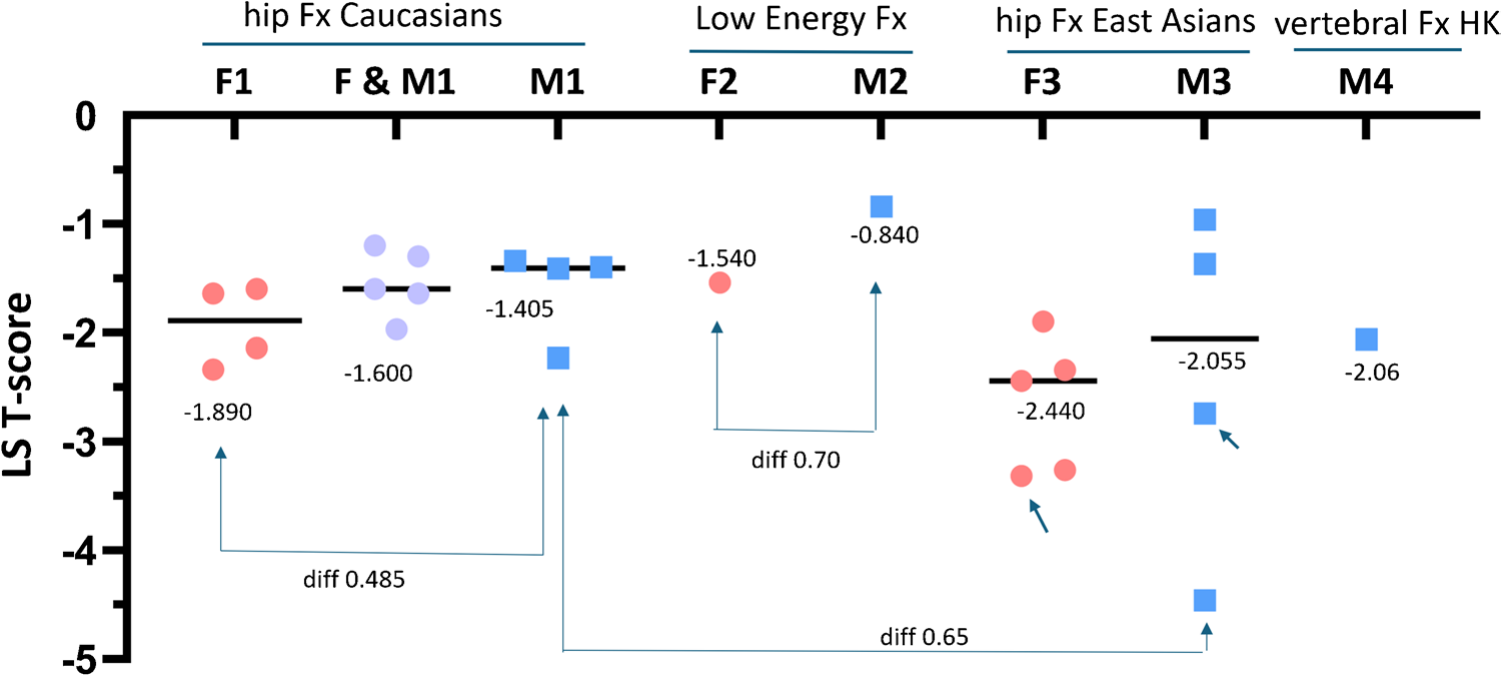
Males suffer from FFx at a higher LS *T*-score than females. Females suffer from FFx at a median T-score higher than the osteoporosis threshold (i.e., LS *T*-score of −2.5) and males suffer from FFx at a median *T*-score higher than the tentative osteofrailia threshold (i.e., LS *T*-score of −2.0). diff: difference. F: females, M: males. Each dot represents one study (sample size unadjusted), and value (black bar) are median values for these listed studies. F&M1: a mixture of males and females. F1: data from Wilson et al. J Bone Joint Surg Br 2009;91:772–5; Yeo et al. Patient Saf Surg 2015;9:39; Schnabel et al. Zentralbl Chir 2005;130:469–75; Olszewski et al. Ortop Traumatol Rehabil 2006;8:395–401. F&M1: data from Amar et al. Arch Osteoporos 2019;14:63; Heetveld et al. J Bone Joint Surg Br 2005;87:367–73; Valentini et al. Nutr Metab Cardiovasc Dis 2020;30:49–55; Houel et al. Osteoporos Int. 2025;36:1061–1068; Marchasson et al. Arch Osteoporos. 2024;19:20. M1: data from Wilson et al.; Yeo et al.; Cesme et al. Acta Orthop Traumatol Turc 2016;50:548–553.; Olszewski et al. For F1, F&M1, and M1, results were of DXA *T*-score measured at the timepoint of a hip FFx. Note that, the median value of the F1&M1 group lies between those of F1 group and M1 group. All included Caucasian data were from Europe or Turkey. F2 and M2: data from Wong et al. Intern Med J. 2003;33:505–10. For F2 and M2 (an Australian study, assumed majority of the study subjects being Caucasians), results were of DXA *T*-score measured within 12 months of the Fx incident. The data of F2 and M2 are patients with low energy trauma fracture. Low energy traumas of the humerus, distal forearm, and spine also commonly occur among subjects with normal bone strength. Therefore, the F2 and M2 values are higher than the F1 and M2 values. F3: data from Li HL et al. BMC Musculoskelet Disord. 2021;22:728; Lee et al. J Bone Metab. 2022;29:51–57. Gani et al. PLoS One. 2020;15:e0241616. Zhu et al. Sci Rep. 2016;6:34185; and data of MsOS Hong Kong study. M3: Data from Li HL et al.; Lee et al. Gani et al. PLoS One. 2020;15:e0241616, and MrOS Hong Kong study. Arrow in F3 and M3: data of Ms. OS and MrOS Hong Kong study till year-3 follow-up (hip FFx incident was approximately 1.5 years after BMD measurement). M4: MrOS Hong Kong study baseline data, mean *T*-score of subjects with OLVFss ≤ − 2.5 (i.e., subjects with osteofrailiac vertebral Fx) and cases with positive LS *T*-score excluded (as these data were contaminated by artifacts)

It has been noted that Caucasian men suffer from FFx at higher FN BMD/*T*-score and higher LS BMD/*T*-score than Caucasian women [2, 33–35]. In Fig. 5, we re-use the systematic literature research results described in [2] and [36], and updated with two recent publications and also our Hong Kong data. Figure 5 shows that, though with limited evidence, hip FFx occur at approximately 0.5 LS *T*-score higher in men (median value: −1.40) than in women (median value: −1.89). Therefore, the same as with the definition of FN osteofrailia, we can define a LS osteofrailia *T*-score cutpoint value to be ≤ − 2.0.

Figure 5 also shows that, on average, hip FFx occur at 0.6 *T*-score higher than the osteoporosis LS cutpoint value (i.e., −2.5) for women, and 0.6 *T*-score higher than the osteofrailia LS cutpoint value (i.e., −2.0) for men, suggesting the difficulty for LS BMD to predict hip FFx.

## At around age 78, if the cutpoint LS QCT BMD of <100 mg/mL is used, half of the Caucasian men are osteofrailiac

**Fig. 6:**
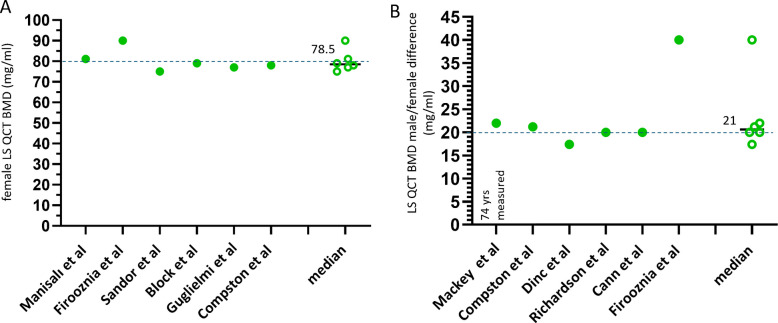
Women’s LS QCT BMD at the age of around 78 years (**A**) and the difference of BMD between men and women (**B**). Data were reported from USA or Europe and Turkey, and it is assumed that the study subjects were predominantly Caucasians. It is noted that, at the age of around 78 years, Caucasian women have a mean BMD of around 80 mg/mL, and men’s BMD is approximately 20 mg/mL higher. Data are from Manisal et al., Clin Orthop Relat Res 2006;443:109–12; Firooznia et al. J Comput Tomogr. 1984;8:91–7; Sandor et al. Calcif Tissue Int 1992;50:502–6; Block et al. J Bone Miner Res 1989;4:249–57; Guglielmi et al. Eur Radiol 1995;5:269–75; Compston et al. Br J Radiol 1988;61:631–6; Mackey et al. J Bone Miner Res 2007;22:1862–8; Dinç et al. Eur J Radiol 1995;21:79–83; Richardson et al. Clin Orthop Relat Res 1985;(195):224–38; Cann et al. Bone 1985;6:1–7. The data of Mackey et al. are measured for 74-year-old subjects, others are fitted data

At the age 78 years old, the mean LS QCT BMD is around 80 mg/mL for Caucasian women, thus at this age half of the Caucasian women are densitometrically osteoporotic by current definition (Fig. 6A). At this age, LS QCT BMD is approximately 20 mg/mL higher for Caucasian men than for Caucasian women (Fig. 6B). If prevalence of Caucasian men with osteofrailia is to be similar as the prevalence of Caucasian women with osteoporosis, then LS QCT BMD of around <100 mg/mL can be used as cutpoint value of osteofrailia for Caucasian men.

## If LS QCT BMD cutpoint value is <100 mg/mL for Caucasian men, the detection sensitivity for FFx in men may be in parallel to the detection sensitivity for FFx in Caucasian women when cutpoint value is <80 mg/mL

**Fig. 7:**
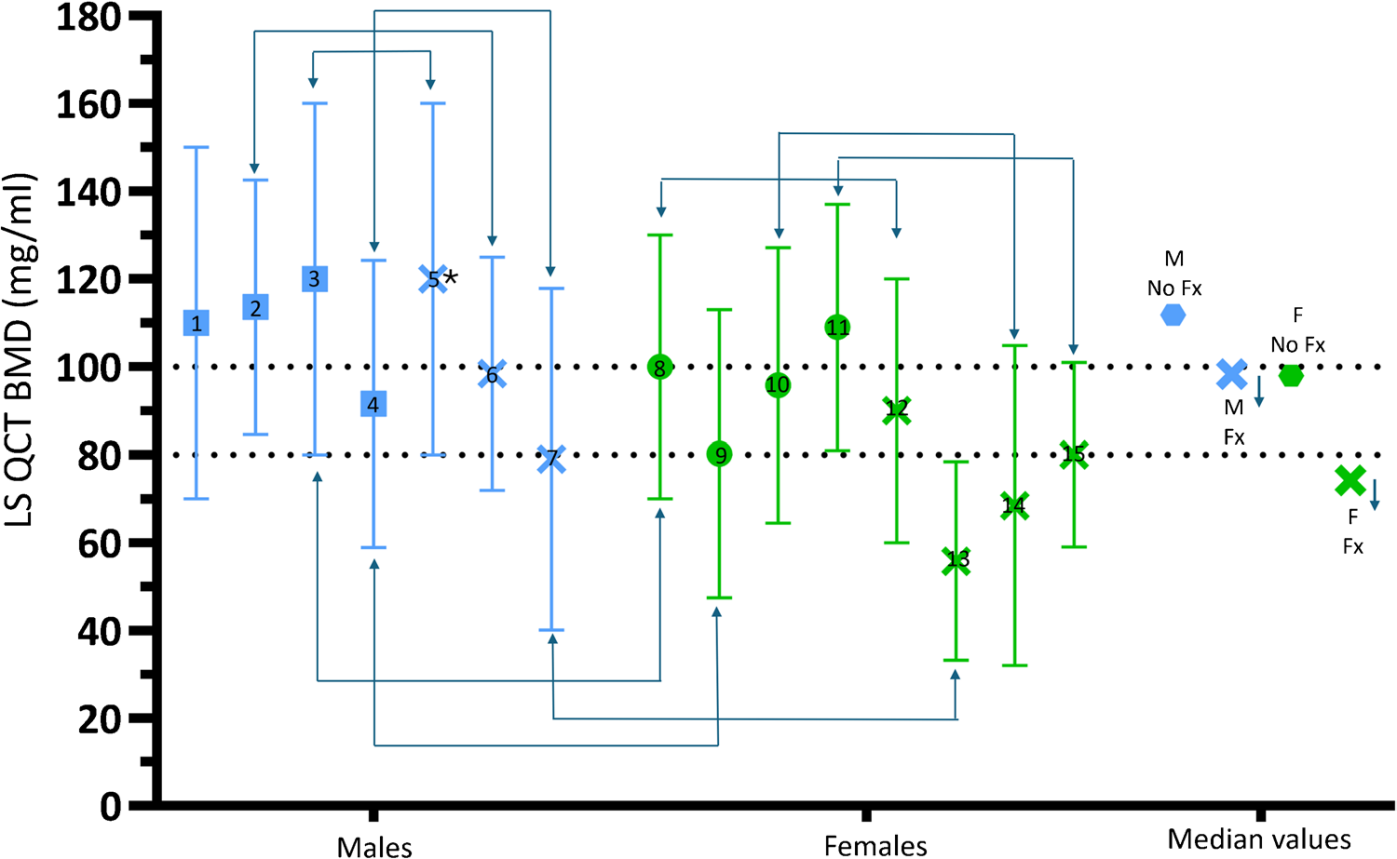
Among older Caucasian populations, male subjects without Fx (M no Fx) have a mean LS QCT BMD higher than 100 mg/mL and female subjects without Fx (F no Fx) have a mean LS QCT BMD higher than 80 mg/mL; male patients with Fx (M Fx) have a mean LS QCT BMB lower than 100 mg/mL and female patients with Fx (F Fx) have a mean LS QCT BMD lower than 80 mg/mL. **×**: patient group with Fx. Blue arrows mean that the BMD would likely be further lower if all the patients were hip FFx patients. *: subjects with Fx during follow-up had the same BMD as those without Fx during follow-up, suggesting the Fx cases might not have been true FFx. 1: data from Chalhoub et al. Bone 2016;92:100–106, males with mean age 73.5 years. 2 and 6: data from Tuck et al. Bone 2022;164:116513. 2: control males without Fx, mean age: 64 years; 6: males with distal forearm fracture, mean age: 63 years. 3, 5, 8, 12: data from Mackey et al. J Bone Miner Res 2007;22:1862–8. 3: no Fx males, mean age: 74.0 years; 5: males with incident non-vertebral fractures during follow-up (mean: 6.4 years), mean age: 75.0 years; 8: no Fx females, mean age: 73.4 years; 12: females with non-vertebral fractures during follow-up (mean: 6.4 years), mean age: 73.8 years. 4, 7, 9, 13: data from Kopperdahl et al. J Bone Miner Res 2014;29:570–80. 4: no Fx males, mean age: 74.8 years; 7: males with incident radiographic vertebral Fx of ≥25% height loss during follow-up (5 years), mean age: 76.5 years; 9: no Fx females, mean age: 74.3 years; 13: females with incident radiographic vertebral Fx of ≥25% height loss during follow-up (5 years), mean age: 76.7 years. 10 (no Fx) and 14 (with Fx): data from Bauer et al. Am J Roentgenol. 2007;188:1294–301, with female of mean age of 71 years; Fx were radiographical Fx. 11 (no Fx) and 15 (with Fx): data from Ito et al. Calcif Tissue Int 1997;61:123–8, with female of mean age of 68 years; Fx were radiographical Fx

In a USA-based study, vertebral FFx threshold was approximately 25 mg/mL higher for men than for women [33]. LS QCT BMD osteofrailia cutpoint of 100 mg/mL might then be reasonable for FFx prediction, as shown in Fig. 7. Among older Caucasian populations, male subjects without fracture (Fx) have a median QCT BMD higher than 100 mg/mL and female subjects without Fx have a median QCT BMD higher than 80 mg/mL; male patients with Fx have a median QCT BMD lower than 100 mg/mL and female patients with Fx have a median QCT BMD lower than 80 mg/mL. For positive and negative FFx patient discrimination, LS QCT BMD of 100 mg/mL in older Caucasian men may be comparable to 80 mg/mL in older Caucasian women.

Figure 5 shows that, the majority of female patients with FFx have a LS DXA *T*-score > −2.5 and the majority of male patients with FFx have a LS DXA *T*-score > −2.0, thus LS *T*-score is not sensitive in predicting FFx. Figure 7 shows the potential that LS QCT has a higher sensitivity in predicting FFx than LS DXA, consistent with data shown in Fig. 4 and the empirical evidence of higher sensitivity QCT for vertebral FFX prediction [30–32].

Additionally, Fig. 7 shows high heterogeneity both for men and for women, and for subjects with and without Fx. This could be due to the heterogeneity in age of the various groups and the heterogeneity in Fx classification. None of the studies in Fig. 7 used the more definitive hip FFx as the clinical endpoint. It is easier to ascertain that a spine fractural deformity in older women is ‘osteoporotic’; while spine fractural deformity is more common and has a higher degree of severity in healthy men [28, 37, 38]. Another possibility is that Caucasians from Northen Europe and Caucasians from Southern Europe may have different normative LS BMD values. This will be discussed in the next section.

As a summary of what has been discussed in sections 6, 7, and 8, we can tentatively recommend that, for Caucasian men, LS osteofrailia *T*-score is ≤ − 2.0 and osteofrailia LS QCT BMD is <100 mg/mL (Table 2).

**Table 2:** observations and results for supporting osteofrailia criterion in Caucasian men

Osteofrailia criterion	Supporting observations and results	Evidence strength
LS DAX *T*-score cutpoints value ≤ − 2.0	Caucasian males hip FFx occur at 0.5 LS *T*-score higher than Caucasian females	modestly strong
	Caucasian males hip FFx occur at 0.5 LS *T*-score higher than East Asian males	weak
	If FN cutpoints value is ≤ −2.0 (rather than −2.5), then LS cutpoints value is ≤ −2.0 (rather than −2.5), this is convenient.	n/a
LS QCT BMD cutpoint value <100 mg/ml	At the age of 78 years, half of the women are osteoporotic when applying a LS QCT BMD <80 mg/ml threshold, half the men are osteofrailiac when applying a LS QCT BMD <100 mg/ml threshold.	modestly strong
	100 mg/ml to separate Caucasian males with FFx and without FFx is comparable to 80 mg/ml in separating Caucasian females with FFx and without FFx.	modestly strong

## Young Caucasian men have a higher DXA BMD heterogeneity than young Caucasian women, and young northern Europeans have a higher DXA BMD and a higher DXA BMD heterogeneity than young southern Europeans

Mediterranean Europeans have better overall bone health than northern Europeans [39–43]. The hip fracture incidence rates are the highest in the Scandinavian countries particularly those of Norway, Denmark, Sweden, and Iceland, and lower among Southern Europeans [40, 41]. Lucas et al. [41] predicted that, the maximum hip fracture incidence rate (per 100,000 subjects) is 1389.8 for Swedish women and 1089.7 for Danish women (742.4 for Swedish men, 551.1 for Danish men), 376.0 for Portuguese women and 420.0 for Spanish women (156.9 for Portuguese men, and 195.0 for Spanish men). Using the European Vertebral Osteoporosis Study (EVOS) data, O’Neill et al. [42] described that radiographic vertebral deformity prevalence was highest in the Scandinavian populations. In the European Prospective Osteoporosis Study (EPOS), Felsenberg et al. [43] described that age-standardized incidence of morphometric vertebral fracture was 17.7 and 7.3 per 1000 person-years for older Scandinavia women and men, and 10.2 and 3.6 per 1000 person-years for older Southern Europeans.

**Fig. 8:**
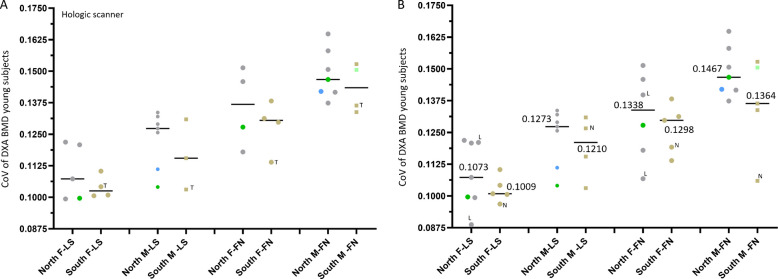
Northen Europe populations have a higher DXA areal BMD heterogeneity than Southern Europe populations, and males have a higher DXA areal BMD heterogeneity than females. Coefficient of variation (CoV) is ‘standard deviation divided by the mean’. **A**: data of Hologic scanner. B: data of mixed Hologic scanner, Lunar/GE scanner (denoted with ‘L’), and Norland scanner (denoted with ‘N’). Each dot represents one study (sample size unadjusted), and value (black bar) are median values for these listed studies. The Northen populations included those in northern Europe, Belgium, France (Montceau-les-Mines, denoted in blue), Australia, Canada, and USA (Caucasians only, denoted in green). The Southern Europe populations included those in Greece, Spain, Italy, Turkey (denoted with letter ‘T’), as well as Iran for femoral neck (denoted in light blue). For a variety of reasons, including differences in X-ray energy generation, bone edge detection algorithms, region of interest placement, and methods of calibration, Lunar scanner is known to derive higher absolute BMD value than Hologic scanner. As A and B show the same trends for CoV, data from all scanners are quantitatively compared among groups **B**. F-LS, female lumbar spine; M-LS, male lumbar spine; F-FN, female femoral neck; M-FN, male femoral neck. Data are from Kudlacek et al. Eur J Clin Invest 2003;33:332–9 (Austria, northern, F-LS, M-LS, F-FN, M-FN); Høiberg et al., Osteoporos Int 2007;18:1504–14 (Denmark, northern, M-LS, M-FN); Kaptoge et al. Bone. 2008;43:332–339 (Belgium, northern, M-LS, M-FN; Portugal, southern, F-LS, M-LS, F-FN, M-FN); Lehmann et al. Calcif Tissue Int. 1995;56:350–4 (Germany, northern, F-LS, M-LS); NHANES III (USA, northern, F-LS, M-LS, F-FN, M-FN); Löfman et al. J Clin Densitom. 2000;3:177–86 (Sweden, northern, F-LS, F-FN); Kröger et al. Osteoporos Int 1992;2:135–40 (Finland, northern, F-LS, F-FN); Tenenhouse et al. Osteoporos Int 2000;11:897–904 (Canada, northern, F-LS, M-LS, F-FN, M-FN); Petley et al. Br J Radiol. 1996;69(823):655–60 (UK, northern, F-LS, F-FN); Szulc et al. Bone. 2000;26:123–9 (France: Montceau-les-Mines, northern, M-LS, M-FN); Henry et al. Osteoporos Int. 2010;21:909–17 (Australia, northern, M-FN); Pedrazzoni et al. Osteoporos Int 2003;14:978–82 (Italy, southern, F-LS, F-FN); Hadjidakis et al. Eur J Clin Invest. 1997;27:219–27 (Greece, southern, F-LS, M-LS, F-FN, M-FN); Diaz Curiel et al. Osteoporos Int 1997;7:59–64 (Spain, southern, F-LS, M-LS, F-FN, M-FN); Manisali et al., Eur Radiol. 2003;13:157–62. (Turkey, southern, F-LS, M-LS, F-FN, M-FN); Larijani et al. J Clin Densitom. 2006;9:367–74 (Iran, southern, M-FN, Hologic scanner value adjusted in Henry et al. Osteoporos Int. 2010;21:909–17)

**Fig 9:**
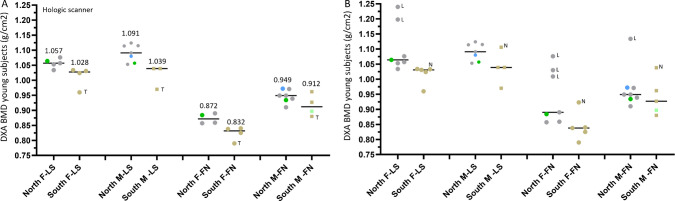
Northen Europe populations have a higher DXA areal BMD than Southern Europe populations. **A**: data of Hologic scanner. **B**: data of mixed Hologic scanner, Lunar/GE scanner (denoted with ‘L’), and Norland scanner (denoted with ‘N’). Each dot represents one study (sample size unadjusted), and value (black bar) are median values for these listed studies. The Northen populations include those in northern Europe, Belgium, France (Montceau-les-Mines, denoted in blue), Australia, Canada, and USA (Caucasians, denoted in green). The Southern Europe populations included those in Greece, Spain, Italy, Turkey (denoted with letter ‘T’), as well as Iranian for femoral neck (denoted in light blue). Data sources are the same as those in Fig. 8., added with the data of Sagelv et al., Arch Osteoporos. 2024;19:58 (where the standard deviation of the results was not available). A and B show the same trends, data from Hologic scanners are quantitatively compared among groups (A)

Considering the substantial difference in FFx prevalence, normative DXA BMD, and QCT BMD among Caucasians and among East Asians (all values being lower among East Asians), we looked into the heterogeneity of European male and female populations, to understand whether there is difference among Northern Europe populations (‘*North’*) and Southern Europe populations (‘*South*’) in normative BMD value, for which only DXA BMD values are available. In our analysis (Fig. 8 and 9), the ‘*North’* included data of Sweden, Norway, Denmark, Finland, Germany, Austria, Belgium, France (Montceau-les-Mines), and also included the data of USA, Canada and Australia. The *‘South’* included Spain, Portugal, Italy, Greece, Turkey, and Iran (male FN only for Iranians). USA, Canada, Australia Caucasians are not ‘*North’* population only, but the data can fit into the *‘North’* group by approximation of the most numerous genetic ancestry/ethnic group for which data are available. As there is limited data of ‘*South*’ populations, data of Turkey and Iran are also included.

Figure 8 shows young Caucasian men have higher DXA BMD heterogeneity than young women. For young subjects, the male-female difference in DXA BMD coefficient of variation (CoV) was 18.6% [=(0.1273-0.1073)/0.1073], 19.9%, 9.64%, 5.08%, respectively (Fig. 8B shows results based on all DXA scanners), for ‘*North*’-LS, ‘*South*’-LS, ‘*North*’-FN, and ‘*South*’-FN, with values on males being higher (i.e., males are more heterogeneous) and particularly so for the LS data. As the *T*-score is calculated as: (BMD_patient_–BMD_young adult mean_)/SD_young adult population,_ due to the larger SD for males, it is more difficult to for males to have ‘very negative’ T-score, and this is even more noticeable for the LS. This can partially explain young male populations having a higher normative T-score, and older men suffering from FFx at higher *T*-score than older women [2]. The ‘*North-South*’ difference in CoV was 6.34% [=(0.1073-0.1009)/0.1009], 5.21%, 3.08%, and 7.55%, respectively, for female LS (F-LS), male LS (M-LS), female FN (F-FN), and male FN (M-FN), with values from ‘*North*’ populations being higher (i.e., ‘*North*’ populations are more heterogeneous).

Figure 9 shows young ‘*North’* populations have a higher DXA BMD than young ‘*South’* populations. The *‘North’-‘South’* difference for DXA BMD among young subjects is 2.28% [=(1.057-1.028)/1.028], 5.0%, 4.81%, and 4.06%, respectively, for F-LS, M-LS, F-FN, and M-FN (Fig. 9A results based on Hologic DXA scanners).

## Limitations and further research directions

There are many limitations for the analyses described in this article. With QCT, multiple technical and acquisition factors, such as calibration precision, CT parameters, the use of single-slice QCT covering multiple vertebrae or volumetric measurement with spiral CT, region of interest (ROI) placement, as examples, need to be taken into account, as these affect the final QCT reading. However, studies have demonstrated that disagreements between QCT measurements are usually minor, if appropriate calibration is regularly conducted. This article further highlights the difficulties of LS DXA *T*-score to predict FFx (Figs. 4 and 5). LS *T*-score measurement is contaminated by degenerative changes which artificially increase LS DXA BMD and *T*-score among older population. Many studies show that, while a stepwise decrease of LS *T*-score is observed following increased age among older women, this stepwise decrease of LS *T*-score is not observed among old men, suggesting a heavier contribution of degenerative changes to *T*-score measurement error among older men than among older women [44, 45]. OLVFss as a parameter is also not as predictive for hip FFx risk in men as in women [3]. We have shown that spine fractural deformity is substantially associated with further radiographic vertebral FFx risk in four years’ time in older Chinese women, but barely so in older Chinese men [46, 47].

**Fig. 10:**
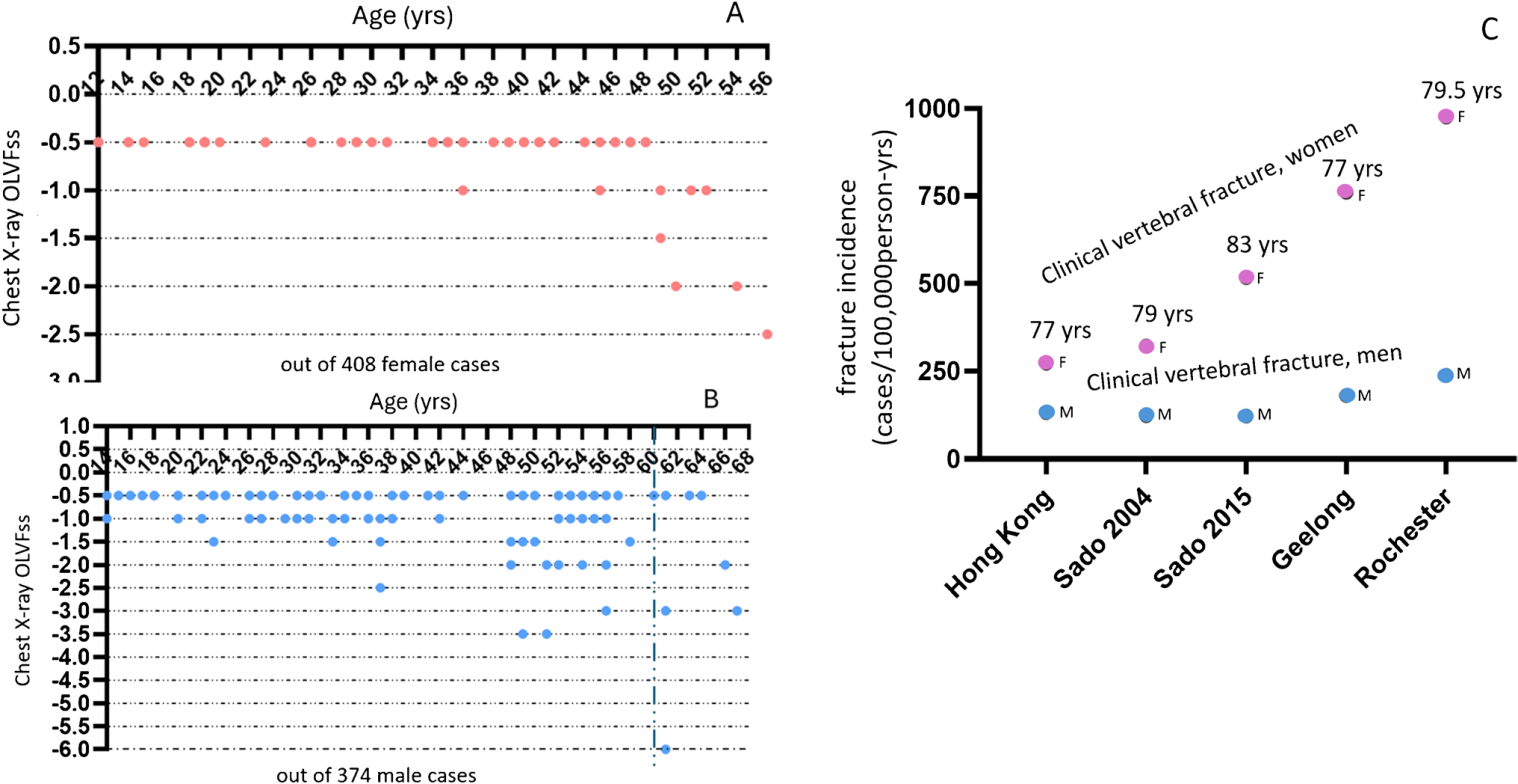
Healthy men have higher prevalence and severity of fractural deformity than healthy women (**A**, **B**), and clinical vertebral Fx are much less common among older men than older women (**C**). **A** and **B**: OVLFss based on lateral chest radiographs of young and middle-aged Chinese women and men without spine or metabolic disorders. OLVFss is calculated by summing up the scores of vertebrae T4 to L1 or L2 (depending on the visibility of L2 on the lateral chest radiograph). Subjects with any OLVF are presented. It can be assumed that for women <44 years and men <60 years, there would be few cases, or none, with osteoporosis. However, the term OLVF is still used here, as these some of the deformities look like osteoporotic fracture. Vertical dotted line shows separation of men <60 years old and ≥ 60 years old. Adapted with permission from Ma and Wang, J Thorac Dis 2022;14:4685–98. C: Incidences of clinical osteoporotic vertebral fracture in older men are mostly less than half of the incidences of clinical osteoporotic vertebral fracture in older women. Data are from Wáng et al., Quant Imaging Med Surg 2022;12:2090–2105 (the Osteoporotic Fracture in Men and Women and Hong Kong studies); Sakuma et al., J Bone Miner Metab 2008;26:373–378 (Sado 2004), Imai et al., J Bone Miner Metab 2019; 37:484–490 (Sado 2015); Sanders et al., Osteoporos Int 1999;10:240–247 (Geelong study); Cooper et al., J Bone Miner Res 1992;7:221–227 (Rochester study). The mean ages in years during the follow-up period for each study are noted. M, male data; F, female data

The arguments in this article are heavily dependent on the initial definition that the LS *T*-score and QCT BMD osteoporosis cutpoint values are ≤ − 2.5 and < 80 mg/mL, respectively, for Caucasian women. For better defining the optimal LS QCT osteofrailia cutpoint value as a biomarker for Caucasian men, an important issue is to define what the clinical endpoint is [48]. Low energy trauma of the humerus, distal forearm, and spine commonly occurs among young subjects with normal bone strength, with approximately one third of fractures among young patients reporting only low energy trauma [49–55]. Hip FFx is the most clinically relevant and most reliable endpoint. However, as noted above, currently the relationship between LS QCT BMD and hip FFx risk is not well established. Identifying high-risk patients for hip FFx among male population is important, as once a hip FFx occurs, the mortality is higher for male patients than for female patients [2, 56]. While vertebral fracture with ≥25% height loss among older women is more likely to be osteoporotic [37, 38], clinical vertebral fractures are much less common among older men than among older women, and vertebral fractures with 1/3 height loss among older men are still less likely to be osteoporotic or osteofrailiac (Fig. 10) [28, 37, 38]. These issues can be particularly problematic when the study sample size is small, which is often the case for FFx clinical studies. This study did not discuss a QCT BMD osteopenia cutpoint value for men, as there is no biological or epidemiological rationale for the threshold of osteopenia [57]. The prevalence of osteopenia according to the common definition is usually too high to be clinically determinant [57].

In conclusion, for the purpose of FFx risk prediction, we propose an osteofrailia (inclusive of osteoporosis) threshold LS DXA *T-*score of ≤ − 2.5 and ≤ −2.0, and QCT BMD of <68 mg/mL and < 100 mg/mL, for East Asian men and Caucasian men, respectively. QCT BMD relates better than DXA *T*-score to FFx risk in men. Our analyses show larger DXA BMD heterogeneity among Caucasian young men than among Caucasian young women, larger heterogeneity among ‘*North’* young populations than among ‘*South’* young populations, and higher DXA BMD among ‘*North’* young populations than among ‘*South’* young populations. Country-specific data should be available in the future, as more normative Caucasian QCT BMD values are being sampled. There is a chance to do this opportunistically, as an example in patients examined for high-energy trauma. The relationship between LS QCT BMD and hip FFx risk should be better investigated in the future.

## Data Availability

N/A, this is a review article.

## Abbreviations

AUROC: Area Under the Receiver Operating Characteristic curve
BMD: Bone mineral density
CoV: Coefficient of variation
DXA: Dual-Energy X-ray Absorptiometry
Fx: Fracture
FFx: Fragility fracture
FN: Femoral neck
LS: Lumbar spine
QCT: Quantitative computed tomography
MrOS: Osteoporotic fractures in men
MsOS: Osteoporotic fractures in women
OLVF: Osteoporotic-like vertebral fractural deformity
OLVFss: Osteoporotic-like vertebral fractural deformity sum score
SD: Standard deviation
